# An Umbrella Review of Meta‐Analyses on the Efficacy and Safety of Eltrombopag in Immune Thrombocytopenia

**DOI:** 10.1002/hsr2.71429

**Published:** 2025-12-14

**Authors:** Mahsa Dabir, Maryam Kamrani Mousavi, Shervin Manteghi, Mohammad Amin Mansoori, Fatemeh Karpasand, Fatemeh Zoghi, Mehrdad Payandeh

**Affiliations:** ^1^ Department of Medical Laboratory Science, School of Paramedical Kermanshah University of Medical Sciences Kermanshah Iran; ^2^ Student Research Committee Kermanshah University of Medical Sciences Kermanshah Iran; ^3^ Student Research Committee Tabriz University of Medical Sciences Tabriz Iran; ^4^ Department of Nutrition Science, Faculty of Nutrition and Food Science Tabriz University of Medical Sciences Tabriz Iran; ^5^ School of Nursing and Midwifery Kermanshah University of Medical Sciences Kermanshah Iran; ^6^ Department of Hematology and Medical Oncology Kermanshah University of Medical Sciences Kermanshah Iran

**Keywords:** eltrombopag, immune thrombocytopenia, meta‐analysis, thrombopoietin receptor agonists, umbrella review

## Abstract

**Background and Aims:**

Immune thrombocytopenia (ITP) is an autoimmune disorder with increased bleeding risk. Eltrombopag, an oral thrombopoietin receptor agonist, is widely used after corticosteroids or immunoglobulins. Multiple meta‐analyses support its efficacy, yet safety concerns persist. We conducted an umbrella review to synthesize evidence on efficacy and safety across age groups.

**Methods:**

We searched PubMed, Scopus, and Web of Science through April 2025 for systematic reviews/meta‐analyses on eltrombopag in ITP. Methodological quality was appraised using AMSTAR 2. Outcomes included platelet response, bleeding, and adverse events. We mapped primary trials across reviews to address overlap and qualitatively summarized findings; no new pooling was performed.

**Results:**

Nine meta‐analyses met inclusion. Across adult/mixed populations, eltrombopag increased platelet response (e.g., RR ≈ 3.4, *p* < 0.001) and reduced bleeding; pediatric effects were directionally similar but less precise with higher heterogeneity. Reported adverse events included hepatobiliary abnormalities and thrombotic events, underscoring the need for monitoring.

**Conclusion:**

Eltrombopag is effective in raising platelet counts and reducing bleeding in ITP. Benefits appear to increase up to ~50 mg daily and plateau thereafter. Age‐related variability and safety considerations support individualized dosing and vigilant follow‐up.

## 1 Introduction

Primary immune thrombocytopenia (ITP) is an autoimmune disorder characterized by a reduced platelet count and an increased tendency to bleed [1]. The condition affects approximately 3.3 individuals per 100,000 people annually. ITP is defined by a platelet count typically falling below 100 × 10⁹/L, which significantly elevates the risk of bleeding due to impaired blood clotting [2].

First‐line treatment options for ITP usually include corticosteroids, intravenous immunoglobulin, and anti‐D immunoglobulin [3, 4]. If these treatments fail, thrombopoietin receptor agonists (TPO‐RAs) are considered as second‐line therapy [3, 5].

Eltrombopag, one of the TPO‐RAs, has demonstrated high response rates in clinical trials, with response rates exceeding 60% [6, 7, 8]. For patients who do not respond adequately to eltrombopag, switching between TPO‐RAs such as romiplostim and eltrombopag may help stabilize platelet counts and/or achieve a satisfactory platelet response [9].

Eltrombopag has also been approved for the treatment of refractory severe aplastic anemia and shows potential for expanding its use to other thrombocytopenic conditions, beyond ITP [10]. In fact, TPO‐RAs as a group (eltrombopag, romiplostim) are increasingly being used for thrombocytopenia irrespective of the underlying cause [11].

A 2016 meta‐analysis concluded that eltrombopag is both a safe and effective treatment for chronic ITP in both adults and children [12]. However, another meta‐analysis highlighted that, while eltrombopag is effective, adverse events remain common. The incidence of any adverse event was reported at 68.3%, with serious adverse events occurring in 7.6% of cases [13].

Moreover, real‐world pharmacovigilance data have shown some life‐threatening adverse effects including hepatobiliary disorders, thrombosis, infections, and skin disorders, highlighting the need for careful monitoring across diverse patient populations [14, 15].

The aim of this umbrella review is to systematically examine and summarize the findings from multiple meta‐analyses regarding the safety and efficacy of eltrombopag in the treatment of ITP. This review seeks to provide a comprehensive evaluation of the therapeutic outcomes of eltrombopag, including its effectiveness in increasing platelet count and its associated adverse events, based on existing meta‐analytic evidence.

## 2 Methods

### 2.1 Study Design

This umbrella review was designed in accordance with established methodological guidelines for conducting overviews of systematic reviews and meta‐analyses. The objective was to summarize the safety and effectiveness of eltrombopag in the treatment of ITP based on the highest level of evidence. Reporting adhered to the Statistical Analyses and Methods in the Published Literature (SAMPL) guidelines and the Preferred Reporting Items for Systematic Reviews and Meta‐Analyses (PRISMA) 2020 statement for overviews of reviews [16, 17].

### 2.2 Search Strategy

A comprehensive literature search was conducted across three major electronic databases: PubMed, Scopus, and Web of Science, utilizing a combination of MeSH terms and free‐text keywords to identify relevant systematic reviews and meta‐analyses published up to April 2025. The search terms were carefully categorized into three groups: Intervention, Condition, and Study type.

The first group, Intervention, included terms such as “Eltrombopag,” “Benzoates,” “Hydrazines,” “Pyrazoles,” “Revolade,” “SB‐497,” and “Promacta.” The second group, Condition, focused on terms like “Idiopathic Thrombocytopenic Purpura,” “immune thrombocytopenia,” and “Autoimmune Thrombocytopenia.” Lastly, the Study type group comprised the terms “Systematic review” and “Meta‐analysis.”

Search strings for each database were as follows: In PubMed, the search was conducted using the string: (“Eltrombopag” [Title/Abstract] OR “Benzoates” [Title/Abstract] OR “Hydrazines” [Title/Abstract] OR “Pyrazoles” [Title/Abstract] OR “Revolade” [Title/Abstract] OR “SB‐497” [Title/Abstract] OR “Promacta” [Title/Abstract]) AND (“Idiopathic Thrombocytopenic Purpura” [Title/Abstract] OR “immune thrombocytopenia” [Title/Abstract] OR “Autoimmune Thrombocytopenia” [Title/Abstract]) AND (“Systematic review” [Title/Abstract] OR “Meta‐analysis” [Title/Abstract]). In Scopus, the search string was: TITLE‐ABS‐KEY (“Eltrombopag” OR “Benzoates” OR “Hydrazines” OR “Pyrazoles” OR “Revolade” OR “SB‐497” OR “Promacta”) AND TITLE‐ABS‐KEY (“Idiopathic Thrombocytopenic Purpura” OR “immune thrombocytopenia” OR “Autoimmune Thrombocytopenia”) AND TITLE‐ABS‐KEY (“Systematic review” OR “Meta‐analysis”). Finally, in Web of Science, the string used was: Topic = (“Eltrombopag” OR “Benzoates” OR “Hydrazines” OR “Pyrazoles” OR “Revolade” OR “SB‐497” OR “Promacta”) AND Topic = (“Idiopathic Thrombocytopenic Purpura” OR “immune thrombocytopenia” OR “Autoimmune Thrombocytopenia”) AND Topic = (“Systematic review” OR “Meta‐analysis”). This structured approach ensured that all relevant studies related to the intervention, condition, and study type were captured in the search results.

### 2.3 Inclusion and Exclusion Criteria

Studies were included if they met the following criteria: (1) they were systematic reviews or meta‐analyses; (2) they evaluated the effects of eltrombopag in patients diagnosed with ITP; and (3) they were published in English. Exclusion criteria were: primary research articles (e.g., randomized controlled trials, cohort studies), narrative reviews, case reports, conference abstracts, animal or in vitro studies, and any articles not involving eltrombopag or not focused on ITP. Two independent reviewers conducted the selection process. Any discrepancies were resolved through discussion and consensus.

### 2.4 Data Extraction

The data from the included studies were extracted using a standardized form. The extracted information included the first author and year of publication, study country, total sample size (*n*), age group, medical history of participants, dosage of eltrombopag (mg), effect size value (risk ratio [RR] with 95% confidence interval [CI]), treatment duration (in days), and the model used for analysis. All data were independently extracted by two reviewers to ensure accuracy and consistency.

### 2.5 Quality Assessment

Methodological quality of included reviews was assessed using the AMSTAR 2 tool. This tool evaluates 16 domains related to the rigor and transparency of systematic reviews. Each review was classified as high, moderate, low, or critically low quality based on the overall rating. Discrepancies between reviewers were resolved through discussion.

### 2.6 Data Synthesis

A qualitative synthesis was performed across included reviews. When meta‐analytical data were available, the pooled effect estimates reported by the original authors were described, including RRs, odds ratios, or mean differences. Variations in findings and limitations of the evidence were also reported. An inherent challenge in umbrella reviews is the potential overlap of primary studies across included meta‐analyses. To mitigate double‐counting, we mapped study identifiers (trial names/first author‐year) across reviews to qualitatively flag overlap and interpreted pooled magnitudes with caution, emphasizing direction and consistency of effects. We did not compute a quantitative overlap metric (e.g., Corrected Covered Area); future umbrellas should incorporate formal overlap analysis.

Subgroup information (e.g., pediatric vs. adult; dose strata) was pre‐specified at the data‐extraction stage as items of interest; however, in the present umbrella review these subgroup findings are reported descriptively as extracted from source reviews and were not re‐analyzed.

### 2.7 Statistical Reporting Conventions and Software

Effect sizes with 95% CIs were reported, prioritizing effect estimates and precision rather than *p*‐values alone. Graphical outputs, including forest, funnel, and risk‐of‐bias plots, were generated using *R* (version 4.3.2; R Foundation for Statistical Computing, Vienna, Austria) with the packages *meta* (v6.2‐1) and *metafor* (v4.4‐0). No additional inferential models were fitted beyond those reported in the source meta‐analyses. All *p*‐values are two‐sided, with *α* = 0.05. Per journal recommendations, *p*‐values < 0.001 are reported as *p* < 0.001; *p*‐values between 0.001 and 0.01 are reported to the nearest thousandth; and *p*‐values ≥ 0.01 are reported to the nearest hundredth. The terms RR, CI, and heterogeneity (*I*²) are used consistently throughout.

## 3 Results

### 3.1 Study Selection

**Figure 1:**
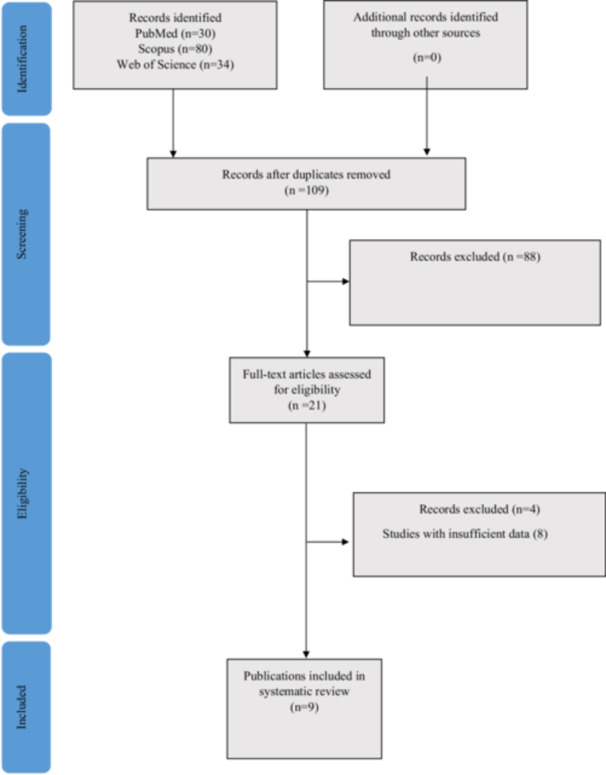
PRISMA 2020 flow diagram showing the process of study selection for the umbrella review.

A total of 144 records were retrieved from the initial search: 30 from PubMed, 80 from Scopus, and 34 from Web of Science. After removing 35 duplicates, 109 records were screened by title and abstract. Of these, 88 were excluded due to irrelevance or failure to meet inclusion criteria. 21 full‐text articles were reviewed, and 9 systematic reviews/meta‐analyses were finally included in the umbrella review. The selection process is illustrated in Figure 1 (PRISMA 2020 Flow Diagram). The methodological quality of these meta‐analyses, assessed using AMSTAR 2, varied from moderate to low, with common limitations including lack of protocol registration, incomplete exploration of heterogeneity, and insufficient reporting of funding sources (Table 1).

**Table 1:** AMSTAR 2 quality assessment of the included meta‐analyses.

AMSTAR questions	Ahmed et al.	Elgebaly et al.	Kolanis et al.	Li et al.	Massaro et al.	Zeng et al.
Did the research questions and inclusion criteria for the review include the components of PICO?	Yes	Yes	Yes	Yes	Yes	Yes
Did the report of the review contain an explicit statement that the review methods were established before the conduct of the review and did the report justify any significant deviations from the protocol?	NR	NR	Yes	Yes	NR	Yes
Did the review authors explain their selection of the study designs for inclusion in the review?	Partially yes	Partially yes	Yes	Yes	Partially yes	Yes
Did the review authors use a comprehensive literature search strategy?	Yes	Yes	Yes	Yes	Yes	Yes
Did the review authors perform study selection in duplicate?	NR	Partially yes	Yes	Yes	No	Yes
Did the review authors perform data extraction in duplicate?	Yes	Yes	Yes	Yes	NR	Yes
Did the review authors provide a list of excluded studies and justify the exclusions?	NR	Yes	Yes	Partially yes	Yes	Yes
Did the review authors describe the included studies in adequate detail?	Yes	Yes	Yes	Yes	Yes	Yes
Did the review authors use a satisfactory technique for assessing the risk of bias in individual studies that were included in the review?	Yes	Yes	Yes	Yes	Partially yes	Yes
Did the review authors report on the sources of funding for the studies included in the review?	NR	NR	Yes	NR	NR	NR
If meta‐analysis was performed did the review authors use appropriate methods for statistical combination of results?	Yes	Yes	Yes	Yes	Yes	Yes
If meta‐analysis was performed, did the review authors assess the potential impact of RoB in individual studies on the results of the meta‐analysis or other evidence synthesis?	No	No	NR	Partially yes	No	Partially yes
Did the review authors account for RoB in individual studies when interpreting the results of the review?	Partially yes	Partially yes	Partially yes	Partially yes	Partially yes	Partially yes
Did the review authors provide a satisfactory explanation for, and discussion of, any heterogeneity observed in the results of the review?	Yes	Yes	Yes	Yes	Yes	Yes
If they performed quantitative synthesis did the review authors carry out an adequate investigation of publication bias and discuss its likely impact on the results of the review?	No	No	NR	Yes	No	NR
Did the review authors report any potential sources of conflict of interest, including any funding they received for conducting the review?	Yes	Yes	Yes	Yes	Yes	Yes

### 3.2 Platelet Response

**Figure 2:**
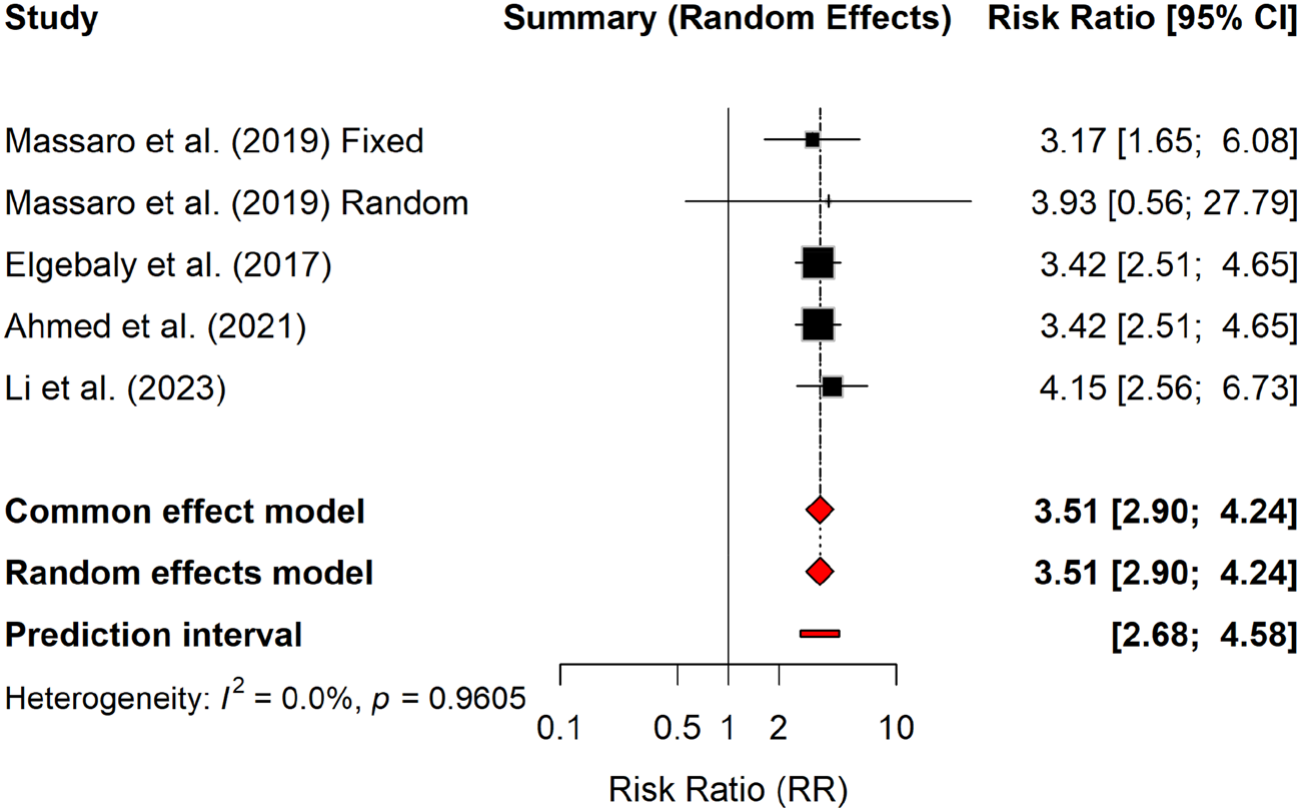
Forest plot showing the relative risk (RR) of platelet response associated with eltrombopag across included studies. Squares represent individual study estimates with 95% confidence intervals (CI), and the size of each square reflects study weight. The diamond indicates the pooled effect using both common effect and random effects models. The prediction interval is shown as a horizontal line at the bottom. Heterogeneity among studies was low (*I*² = 0.0%, *p* = 0.9605).

Eltrombopag demonstrated overall improved platelet response across studied populations. In pediatric chronic immune thrombocytopenic purpura, Massaro et al. (2019) reported no statistically significant improvement in platelet counts with Eltrombopag versus placebo in the random‐effects model (RR = 3.93, 95% CI: 0.56–27.79, *p* = 0.17), though substantial heterogeneity (*I*² = 74%, *p* = 0.05) suggested variability in treatment response [18]. The wide CI and lack of statistical significance suggest that eltrombopag's efficacy in children may lead to variable outcomes. In contrast to pediatric studies, meta‐analyses focused on adult or mixed‐age populations consistently reported a robust platelet enhancement (fixed‐effects model RR = 3.42, 95% CI: 2.51–4.65, *p* < 0.001) with low heterogeneity (*I*² = 22%) [12, 13]. Li et al. (2023) confirmed these findings in adults (random‐effect RR = 4.15, 95% CI: 2.56–6.73, *p* < 0.001) despite moderate heterogeneity (*I*² = 33%) [19]. These results suggest that while pediatric responses may be less predictable, eltrombopag reliably improves platelet counts in mixed and adult populations. Details are summarized in Table 2, and a forest plot illustrating the relative risk values for platelet response across the included studies is shown in Figure 2.

**Table 2:** Summary of included studies reporting the effect of eltrombopag on platelet activity.

Study	Country	Total sample size (*n*)	Age group	Medical history	Dosage (mg)	Effect size value (RR – 95% CI)	Treatment duration (days)	Model
Massaro et al. (2019)	China	159	Children	Chronic ITP	37.5	3.17 [1.65, 6.08]	23	Fixed
	China	159	Children	Chronic ITP	37.5	3.93 [0.56, 27.79]	23	Random
Elgebaly et al. (2017)	Egypt	611	Adults and children	Chronic immune thrombocytopenia	42.7	3.42 [2.51, 4.65]	10.3	Fixed
Ahmed et al. (2021)	China	600	Adults and children	Chronic immune thrombocytopenia	42.7	3.42 [2.51, 4.65]	10.3	Fixed
Li et al. (2023)	China	595	Adults	Persistent/chronic ITP	37.5	4.15 [2.56, 6.73]	—	Random

*Note:* All effect sizes are risk ratios.

Abbreviations: ITP, Immune thrombocytopenic purpura; RR, risk ratio.

### 3.3 Incidence of Bleeding Events

Eltrombopag lowered the incidence of bleeding across all studied populations. Massaro et al. (2019) reported reduced bleeding incidence in the eltrombopag group compared to controls (RR = 0.50, 95% CI: 0.29–0.87; *p* = 0.01), despite persistent heterogeneity (*I*² = 63%) [18]. Findings from subsequent studies further validated this. Another meta‐analysis reported that eltrombopag reduced significant bleeding (fixed‐effects model RR = 0.56, 95% CI: 0.41–0.77; *p* < 0.001), with no heterogeneity (*I*² = 0%). It also reduced the incidence of any bleeding (RR = 0.74, 95% CI: 0.66–0.83; *p* < 0.001), with initial heterogeneity resolved after excluding one pediatric study. Notably, children experienced a more pronounced reduction in bleeding events (RR = 0.51, 95% CI: 0.37–0.71; *p* < 0.0001; *I*² = 63%) compared to adults (RR = 0.83, 95% CI: 0.73–0.93; *p* = 0.002; *I*² = 0%), with significant subgroup differences (*I*² = 85.8%; *p* = 0.008) [12].

A 45 mg dose of eltrombopag significantly lowered bleeding incidence in adults and children with immune thrombocytopenia (fixed‐effect model RR = 0.74, 95% CI: 0.62–0.89; *p* = 0.001), with moderate heterogeneity (*I*² = 68%) [20]. This finding aligns with Zeng et al. (2011), who observed a similar reduction in adults with chronic ITP (fixed‐effects model RR = 0.78, 95% CI: 0.68–0.89; *p* < 0.001; *I*² = 74%) [21].

The 37.5 mg dose previously associated with improved platelet response in adults also reduced bleeding incidence in children (random‐effect RR = 0.50, 95% CI: 0.29–0.87; *p* = 0.01; *I*² = 63%) [19]. Ahmed et al. (2021) initially reported comparable results (RR = 0.65, 95% CI: 0.48–0.87; *p* = 0.003), though with substantial heterogeneity (*I*² = 75%; *p* = 0.001). The effect remained significant after excluding the outlier study (RR = 0.75, 95% CI: 0.60–0.93; *p* = 0.008) [13].

**Figure 3:**
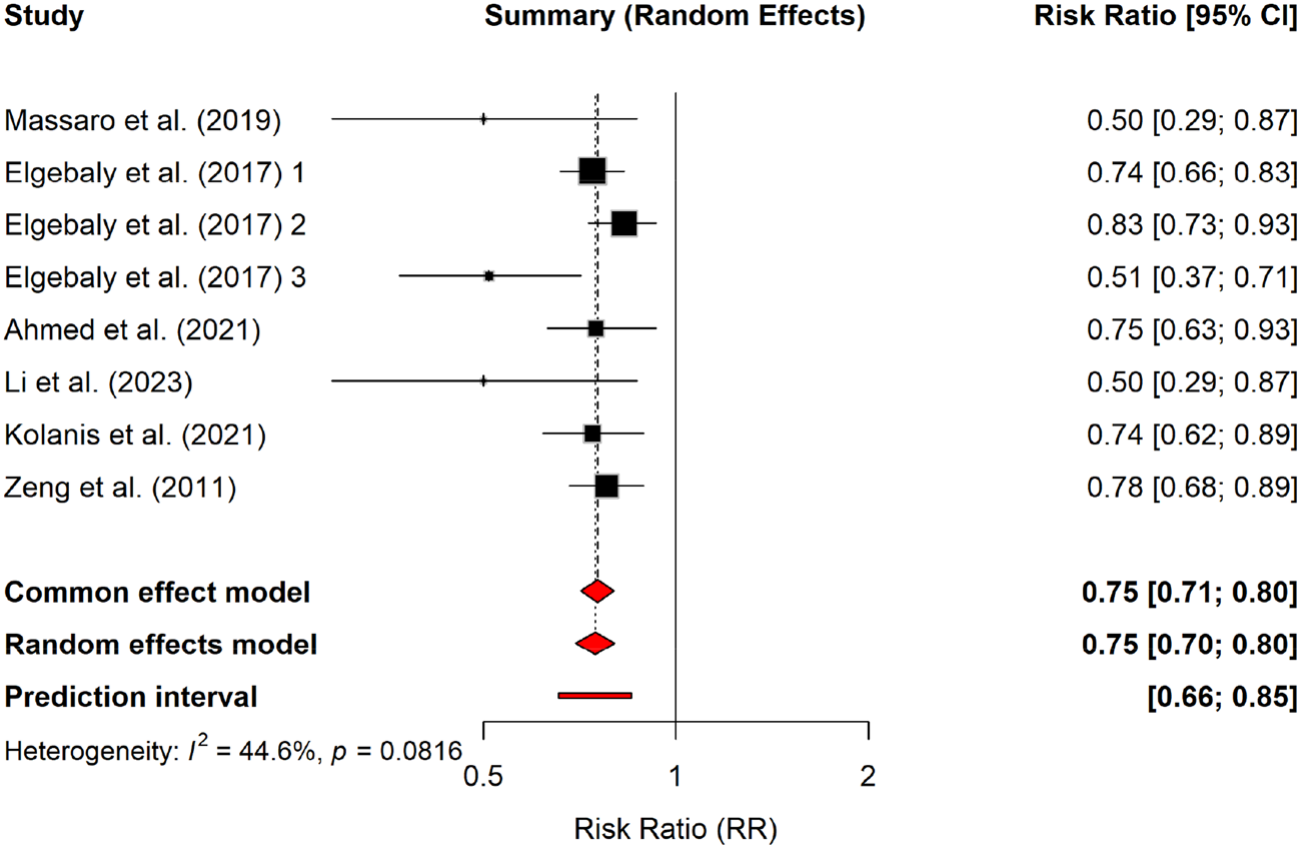
Forest plot showing the relative risk (RR) of bleeding events associated with eltrombopag across included studies. Squares represent individual study estimates with 95% confidence intervals (CI), and the size of each square reflects study weight. The diamond indicates the pooled effect using both common effect and random effects models. The prediction interval is shown as a horizontal line at the bottom. Heterogeneity among studies was moderate (*I*² = 44.6%, *p* = 0.0816).

These results suggest that higher doses of eltrombopag yield more consistent effects across populations compared to the 37.5 mg dose. Taken together, these findings highlight the age‐dependent variations in both the magnitude and consistency of eltrombopag's effects on bleeding outcomes. Pediatric patients appear to benefit from a greater relative reduction in bleeding events, though variability remains higher compared to adults. A summary of findings is provided in Table 3, and a forest plot illustrating the relative risk values for bleeding events across the included studies is shown in Figure 3.

**Table 3:** Summary of included studies reporting the effect of eltrombopag on the incidence of bleeding events.

Study	Country	Total sample size (*n*)	Age group	Medical history	Dosage (mg)	Effect size value (RR – 95% CI)	Treatment duration (days)	Model
Massaro et al. (2019)	China	159	Children	Chronic ITP	37.5	0.5 [0.29, 0.87]	23	Random
Elgebaly et al. (2017)	Egypt	588	Adults and children	Chronic immune thrombocytopenia	45	0.74 [0.66, 0.83]	11.2	Fixed
	Egypt	429	Adults	Chronic immune thrombocytopenia	50	0.83 [0.73, 0.93]	12	Fixed
	Egypt	159	Children	Chronic immune thrombocytopenia	37.5	0.51 [0.37, 0.71]	10	Fixed
Ahmed et al. (2021)	China	666	Adults and children	Chronic immune thrombocytopenia	45.3	0.75 [0.63, 0.93]	11	Random
Li et al. (2023)	China	159	Children	Persistent/chronic ITP	37.5	0.5 [0.29, 0.87]	—	Random
Kolanis et al. (2021)	Greece	625	Adults and children	Immune thrombocytopenia	45	0.74 [0.62, 0.89]	7.4	Fixed
Zeng et al. (2011)	China	327	Adults	Chronic ITP	50.83	0.78 [0.68, 0.89]	6	Fixed

*Note:* All effect sizes are risk ratios.

Abbreviations: ITP, Immune thrombocytopenic purpura; RR, risk ratio.

## 4 Discussion

Eltrombopag is a valuable treatment option for individuals with ITP, as it effectively enhances platelet response and reduces the occurrence of bleeding [22]. However, variations exist in study design, outcome measurement, and the characteristics of the populations can influence the interpretation of results [23]. To provide a comprehensive understanding, this study will integrate data from multiple sources, including systematic reviews and meta‐analyses. Pooled data from randomized controlled trials demonstrated that eltrombopag significantly increased the proportion of adults achieving platelet counts ≥ 50 × 10⁹/L (RR = 3.42; 95% CI: 2.51–4.65), with an overall response rate of approximately 67% across included studies [13]. These results align with the phase III RAISE trial, where a notable percentage of patients maintained consistent platelet responses for at least 75% of their visits [22]. However, when examining pediatric populations, the effectiveness of eltrombopag was found to be lower, potentially because of differences in how the drug is processed in children and the need for more rescue therapy [24]. Some factors can predict a patient's response to treatment; for example, individuals with baseline platelet counts below a specific threshold tend to exhibit greater responses. Also, patients who have had their spleen removed respond similarly to those who have not [25]. In cases where patients do not respond well to the standard dose, increasing it can help maintain target platelet counts in some instances. Data from the included studies suggest that eltrombopag demonstrates a clear dose response effect up to 50 mg daily; however, increasing the dose beyond 50 mg results in a plateau in efficacy as it yields only marginal additional benefit for most patients. It is worth noting that a meta‐analysis highlighted a connection between platelet responses and a reduction in bleeding. Patients who achieved higher platelet counts were less likely to experience significant bleeding [13]. However, this relationship was less clear in studies that did not use standardized bleeding scales.

Eltrombopag has been shown to decrease the occurrence of significant bleeding. However, there is substantial variability among studies, which makes interpreting the results challenging [23]. studies, including phase 2 and phase 3 trials (TRA100773A, TRA100773B, RAISE, REPEAT, and EXTEND), showed that bleeding rates among eltrombopag‐treated patients decreased markedly from baseline levels of 50%–73% to 26%–39% by Week 2, with this reduction sustained throughout treatment. In contrast, placebo‐treated patients showed no such improvement. Specifically, the odds of any bleeding were reduced by 51% to 76%, and clinically significant bleeding was lowered by 65% in eltrombopag‐treated patients compared to placebo (*p* < 0.001) [26]. Different bleeding scales can produce varying results, and open‐label extension trials tend to overestimate effectiveness compared to double‐blind randomized controlled trials [23]. Additionally, the protocols for rescue therapy and the specific characteristics of the patient population can also influence the outcomes. When certain trials are excluded, the variability among studies is resolved, while the significance is maintained. The safety profile of eltrombopag involves balancing its effectiveness with potential risks. Regarding hepatobiliary complications, elevated transaminase levels occur in some patients. Starting with a lower dose can reduce the risk of liver‐related issues [25]. Thrombotic events occur in some patients, and these events often occur when platelet counts exceed a certain threshold, so it's crucial to maintain platelet counts within a safe range. Long‐term use of eltrombopag can lead to bone marrow fibrosis and cataracts, necessitating regular monitoring and ophthalmologic evaluations [23]. When comparing eltrombopag to other treatments, network meta‐analyses suggest that eltrombopag is a viable first‐line alternative to certain procedures. While eltrombopag has comparable efficacy to other treatments, its administration may improve patient adherence, though the costs remain considerable [23].

When compared with other TPO‐RAs, eltrombopag generally shows similar efficacy to romiplostim and avatrombopag in achieving target platelet counts, but it offers the convenience of oral administration compared to the subcutaneous route for romiplostim [27]. Avatrombopag has a more favorable food interaction profile than eltrombopag, potentially improving adherence [28]. However, differences in cost, availability, and patient comorbidities should guide individualized selection among these agents [29].

Because many included systematic reviews and meta‐analyses aggregate multinational evidence without reporting the country mix of the primary studies, geography‐specific inferences are limited. Nevertheless, real‐world data from lower‐ and middle‐income settings align with our pooled conclusions. For example, cohorts from India and Pakistan have reported clinically meaningful platelet responses to eltrombopag in routine practice [30, 31]. Chinese pediatric series (including long‐term and multicenter experiences) support effectiveness and manageable safety in children [32]. These complementary data suggest that the benefits of eltrombopag are not restricted to high‐income countries, although more region‐specific research is warranted.

There are several limitations and areas where more research is needed. These include the lack of standardized bleeding scales, limited data on certain populations, and the need for longer‐term studies. Future research should focus on identifying factors that can predict a patient's response to the drug, conducting registries to track long‐term complications, and comparing the cost‐effectiveness of eltrombopag to other emerging treatments. In conclusion, eltrombopag is a key treatment option for chronic ITP, demonstrating effectiveness in increasing platelet counts and reducing bleeding risk across diverse populations. However, careful monitoring for potential complications and personalized dosing strategies are essential. Future studies should prioritize standardized endpoints, long‐term safety surveillance, and comparisons with newer treatments to optimize patient care [22].

## 5 Conclusion

In conclusion, eltrombopag has emerged as an effective and generally well‐tolerated therapeutic option for patients with ITP, consistently demonstrating the ability to increase platelet counts and reduce the incidence of bleeding events across diverse patient populations and clinical settings. While pediatric patients may show somewhat lower response rates, eltrombopag remains a valuable option, particularly for those who are refractory to first‐line therapies.

## Author Contributions

**Mahsa Dabir:** data curation, methodology, project administration, resources, software, visualization, writing – original draft, writing – review and editing. **Maryam Kamrani Mousavi:** methodology, visualization, writing – original draft, writing – review and editing. **Shervin Manteghi:** writing – original draft, writing – review and editing. **Mohammad Amin Mansoori:** project administration, writing – original draft, writing – review and editing. **Fatemeh Karpasand:** writing – original draft, writing – review and editing. **Fatemeh Zoghi:** writing – original draft, writing – review and editing. **Mehrdad Payandeh:** methodology, project administration, software, supervision, writing – original draft, writing – review and editing. All authors have read and approved the final version of the manuscript.

## Ethics Statement

The authors have nothing to report.

## Consent

The authors have nothing to report.

## Conflicts of Interest

The authors declare no conflicts of interest.

## Transparency Statement

The lead author, Mehrdad Payandeh, affirms that this manuscript is an honest, accurate, and transparent account of the study being reported; that no important aspects of the study have been omitted; and that any discrepancies from the study as planned (and, if relevant, registered) have been explained.

## Data Availability

The data used to support the findings of this study are available from the corresponding author upon request. Mehrdad Payandeh had full access to all data in this study and takes complete responsibility for the integrity of the data and the accuracy of the data analysis.
